# Effect of Race and Dementia Prevalence on a COVID-19 Infection Control Intervention in Massachusetts Nursing Homes

**DOI:** 10.1093/geroni/igab046.2704

**Published:** 2021-12-17

**Authors:** Alyssa Dufour, Cyrus Kosar, Vincent Mor, Lewis Lipsitz

**Affiliations:** 1 Hebrew SeniorLife, Boston, Massachusetts, United States; 2 Brown University, Providence, Rhode Island, United States

## Abstract

Nursing home (NH) residents, especially those who were Black or had dementia, had the highest infection rates during the COVID-19 pandemic. A 9-week COVID-19 infection control intervention in 360 Massachusetts NHs showed that adherence to an infection control checklist with proper PPE use and cohorting, was associated with declines in weekly infection rates. NHs were offered weekly webinars, answers to infection control questions, resources to acquire personal protective equipment, backup staff, and SARS-CoV-2 testing. We asked whether the effect of this intervention differed by racial and dementia composition of the NHs. Data were obtained from 4 state audits using infection control checklists, weekly infection rates, and Minimum Data Set variables on race and dementia to determine whether adherence to the checklist competencies was associated with decline in average weekly rates of new COVID-19 infections. Using a mixed effects hurdle model, adjusted for county COVID-19 prevalence, we found that the overall effect of the intervention did not differ by race, but proper cohorting of residents was associated with a greater reduction in infection rates among facilities with ≥20% non-whites (n=83). Facilities in the middle (50-61%; n=116) and upper (>61%; n=118) tertiles of dementia prevalence had the largest reduction in infection rates as checklist scores improved. Cohorting was associated with greater reductions in infection rates among facilities in the middle and upper tertiles of dementia prevalence. Thus, adherence to proper infection control procedures, particularly cohorting, can reduce COVID-19 infections, even in facilities with high percentages of high-risk residents (non-white and dementia).

